# Internationalization of the Moroccan Journal of Chemistry: A bibliometric study

**DOI:** 10.1016/j.heliyon.2023.e15857

**Published:** 2023-05-12

**Authors:** Hanae Lrhoul, Houcemeddine Turki, Belkheir Hammouti, Othman Benammar

**Affiliations:** a School of Information Sciences, Mohammed V University of Rabat, Rabat, Morocco; b Data Engineering and Semantics Research Unit, Faculty of Sciences of Sfax, University of Sfax, Sfax, Tunisia; c Laboratory of Applied Analytical Chemistry, Materials and Environment (L2ACME), Faculty of Sciences, Mohammed First University, Oujda, Morocco; d Applied Mathematics and Computing Laboratory, Higher Normal School, Hassan II University of Casablanca, Casablanca, Morocco

**Keywords:** Bibliometrics, Internationalization, Scholarly journals, Research community

## Abstract

In this research paper, we analyzed the bibliographic data of the research publications issued by the Moroccan Journal of Chemistry between 2013 and 2021. As an open-access country-based research journal with a narrow area of interest and international online exposure, it will be interesting to see how it affects the local chemical research community through the comparison of the characteristics of the research outputs of the journal as retrieved from the *Directory of Open Access Journals* (DOAJ) with the features of Moroccan chemical research from 2014 to 2021 in the *Web of Science Core Collection* (WoS). In this context, we generated scientometric networks using *Gephi*, a tool for large-scale data visualization, to reveal the patterns of the publications in the Moroccan Journal of Chemistry. When performing our analysis, we found a significant alignment between the research topics featured in the Moroccan Journal of Chemistry and the main research areas of the Moroccan chemical scholarly outputs, particularly Multidisciplinary Chemistry, Physical Chemistry, and Analytical Chemistry. We also identified that the Moroccan Journal of Chemistry functions as an incubator for establishing new traditions of research collaboration between Moroccan institutions and target nations such as Asian and African countries. As well, it is clear that the Moroccan Journal of Chemistry is an interesting venue for the most productive chemical researchers in Morocco for sharing preliminary research findings and discussing trendy topics.

## 1 Introduction

For decades, scholarly journals have been featured as the major means for disseminating research findings and evolving communication between major scientific communities [1]. In Africa, a few scholarly journals have more than a century of history such as *Transactions of the Royal Society of South Africa* (1877, South Africa), *South African Medical Journal* (1884, South Africa), *South African Journal of Geology* (1896, South Africa), *South African Journal of Science* (1905, South Africa), and *La Tunisie Médicale* (1903, Tunisia). With the rise of the digital age, more research journals are being founded across the continent, mainly in South Africa [2], Nigeria [3], Egypt, Morocco, and Algeria [4]. Most of these newly created venues and even some of the oldest journals in Africa such as *La Tunisie Médicale* [5] have chosen to go open-access for better online visibility and an enhanced citation impact [4,6]. Some of these journals have been successfully indexed in the *Directory of Open-access Journals* (DOAJ), a community-curated database of trustworthy open-access research journals created in 2003 and available at https://doaj.org [7]. According to the *DOAJ* database as of August 20, 2022, South Africa is the most represented country with 125 scholarly journals followed by Egypt (63 journals), Morocco (28 journals), Algeria (25 journals), and Nigeria (17 journals).

Five years earlier, the situation was a bit better with 490 indexed journals from Egypt, 72 journals from South Africa, 38 journals from Nigeria, and 8 journals from Morocco [6]. The reason behind this is that multiple scholarly journals have adopted predatory behaviors to have less-experienced authors and audiences favoring quantity at the expense of quality of contributions and readership and this negatively affected their reputation instead of increasing impact and reach leading to their elimination from the journal database [3,8]. By contrast, confirmed African open-access scholarly journals have successfully persisted in DOAJ thanks to various ethical approaches. On the one hand, journals have chosen to use English as their main language to reach the worldwide research community using the first living language currently in use for scientific purposes [3]. On the other hand, several journals such as the *Moroccan Journal of Chemistry* have decided to make publication services free of charge for authors and enhance the indexation of their outputs in highly recognized citation indexes such as *Web of Science* and *Scopus* so that the scientific community can consider them as reliable sources for references and interesting targets for publication.^1^

In this research paper, we will analyze the bibliographic data of the publications in the *Moroccan Journal of Chemistry*, one of the journals issued in the African continent that has limited traditions in open-access publishing, between 2013 and 2021 to verify whether this journal has succeeded in its quest of internationalization and impact. This will be a reliable proof of whether the non-predatory free-of-charge open-access publishing model adopted by several African journals to increase their reach and reputation is efficient or not. For the sake of our analysis, we will restrict our internationalization assessment to the study of the worldwide contributions to the Moroccan Journal of Chemistry. As well, we will study the impact of the national journal on local chemical research through the comparison of the output and country contributions of the journal with the reported changes in Moroccan chemical research. We will begin by providing a literature review of the bibliometric studies that have been conducted to analyze open-access journals, particularly in the Global South (Section 2). Then, we will explain the methods used to analyze the outputs of the Moroccan Journal of Chemistry and compare them with the Moroccan chemical research as revealed by the *Web of Science Core Collection*, a large bibliographic database maintained by Clarivate Analytics (Section 3). After that, we will provide the results of our assessment of the journal and discuss them by comparing them to the status of the local chemical research in Morocco from 2014 to 2021 and previous findings about open-access publishing in Africa and abroad (Section 4). Finally, we will conclude this research work and provide future directions for developing its findings (Section 5).

## 2 Literature review

Scientometrics, or the science to measure and analyze science, is a prominent tool to manage and evaluate scientific research and build national research policy. The main objective of scientometrics is to analyze literature data to collect, manipulate, interpret, and forecast a variety of characteristics such as the performance and the evolution of science [9]. One of the facets of studying research productivity is the assessment of scholarly journals [1]. Scholarly journals are important venues for worldwide research outputs, particularly related to hot topics like COVID-19 [10] and Bioeconomy [11]. That is why their analysis can bring insights into readership behaviors [12], citation-based influence [13], topical coverage [14], and research productivity [6]. Surprisingly, most such assessments are applied to Asian and African journals for searching paths to evolve their research quality and reach in the short run despite the challenges of maintaining them by developing countries in the two continents [1]. This is of major importance as the number of national research journals is highly correlated with the research productivity of every country [15].

With the rise of open-access publishing in developing nations, a recent trend of assessing the outputs of African and Asia open-access journals has been launched to study their effect on local research landscapes and global research productivity [3,16]. These studies cover the analysis of journals from the Middle East [16], Nigeria [3], South Africa [6], and North Africa [14] using a range of techniques including Natural Language Processing, Bibliometrics, and Altmetrics. These studies are sometimes restricted to single nationwide research journals such as *La Tunisie Médicale* [14], *Libyan Journal of Medicine* [17], *South African Journal of Botany* [18], and *Journal of King Saud University – Science* [19]. These research works also include the characterization of new initiatives for preparing the creation of new national open-access journals in Sub-Saharan Africa to increase the visibility of local research in countries like Kenya [20] and Ghana [21]. This implies the study of the perception of the local research community of the importance of launching national open-access research journals [20,21]. Among the factors studied by these outputs, the most important ones are the study of the evolution of the citation impact coverage and reach of the journals and their quest for internationalization from the perspective of citations, contributions, Editorial Board, and readership. By internationalization, we mean the increase of diversity of the geographical distribution for citations, contributions, Editorial Board, and readership [22]. Despite the value of citation, editorial board, and readership data, most internationalization studies mainly emphasized the analysis of the contributions of different countries to nationwide research journals [23,24].

In this research paper, we continue this effort for identifying the patterns of African open-access scholarly journals through the analysis of the *Moroccan Journal of Chemistry*, a free-of-charge open-access journal created in 2013 and issued by Mohammed First University, Morocco. The publishing model of the journal is based on the free and open-source *Open Journal Systems* (OJS) software maintained by the Public Knowledge Project and allowing the low-cost web hosting of online open-access journals [25]. OJS-based journals, including the *Moroccan Journal of Chemistry*, have a transparent peer-review management system mostly led by volunteer editorial board members and immediately publish accepted research papers under the CC-BY-SA 4.0 License without any article processing fees [25]. As of June 24, 2021, Morocco has the second-highest number of OJS-based journals in Africa with 102 journals, mainly maintained by local public universities and hosted by the Moroccan Institute of Scientific and Technical Information (IMIST).^2^ These journals are edited by local scientists with the collaboration of very small international advisory boards and some of them have even reached indexation by major bibliographic databases, particularly *Web of Science* and *Scopus*.

Here, we emphasize whether the publishing model of the Moroccan Journal of Chemistry and consequently of the OJS-based journals leads to the internationalization of the contributions of countries to national research journals and whether the output of such venues influences the local research community.

## 3 Methods

To conduct our analysis, we retrieve all the bibliographic data of the papers published in the *Moroccan Journal of Chemistry* between 2013 and 2021 from the *Web of Science Core Collection* as an Excel spreadsheet (Retrieved on May 4, 2022). These data include the year of publication of the outputs, the full names and countries of the published authors, and the *Keywords Plus* of the publications. *Keywords Plus* are controlled keywords assigned by the *Web of Science Core Collection* database to the indexed research publications through the extraction of noun phrases from the titles of their references. The advantage of these keywords is the assignment of the same term for the same concept across the Web of Science Core Collection database allowing a less granular and more precise topic analysis [26]. After the data is retrieved, we use *Gephi*, a data visualization tool that generates networks from pre-processed datasets [27], to construct scientometric networks describing the productivity outlines of the *Moroccan Journal of Chemistry*: the keyword co-occurrence network and the collaboration networks for published authors and countries. We use default settings to generate the networks and we assign weights to nodes and edges according to their respective total link strength.

After that, to allow the discussion of our research findings, we retrieve statistics about the bibliographic information related to Moroccan chemical research through a simple query of *Web of Science Core Collection*^3^ (Retrieved on August 19, 2022). We cover two periods: 2014–2017 when the journal has been created (1063 publications) and 2018–2021 when the journal became a confirmed research venue (2657 publications). We excluded 2013 from our analysis of Moroccan chemical research because we needed the two compared periods to include the same number of years and because it is not likely that the journal will affect the Moroccan research productivity in a month, particularly as the *Moroccan Journal of Chemistry* has been founded in December 2013.

The *Web of Science Core Collection* data will allow us to characterize how the scholarly journal impacts local chemical research in Morocco. The retrieved data covers the most published authors, the main *Web of Science Categories*, and the most important country collaborations of Moroccan chemical research. *Web of Science Categories* are precise mappings of scholarly research publications indexed in the *Web of Science Core Collection* to the subfields of confirmed research areas. It currently includes 254 subject areas representing all the areas of scholarly research ranging from arts and humanities to management and engineering [28]. The analysis of these categories will be useful to identify whether the topical coverage of the *Moroccan Journal of Chemistry* corresponds to the main research trends of Moroccan chemical research or not.

## 4 Results and discussion

**Fig. 1:**
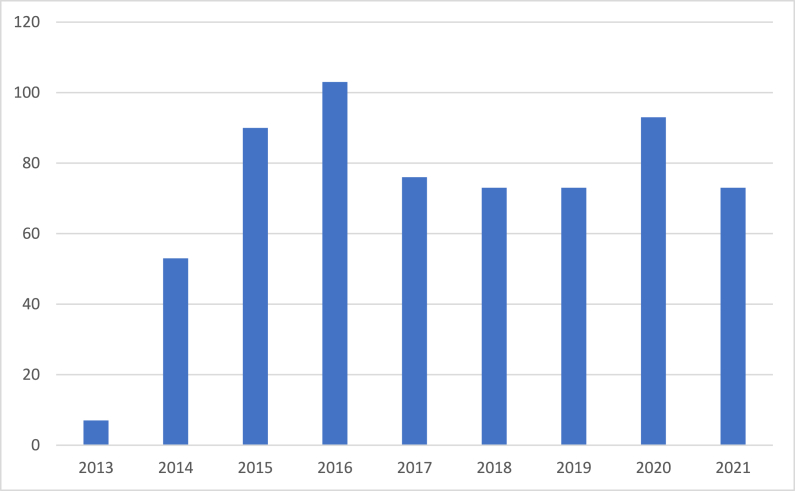
Distribution of the research papers of the Moroccan Journal of Chemistry per publication year.

When retrieving the bibliographic data of the research papers issued by the Moroccan Journal of Chemistry, we have successfully identified 641 papers proving that the journal has been successful to acquire a confirmed community of authors. All the papers are either journal articles or reviews. The distribution of the publications per year shown in Fig. 1 proves that the journal has achieved an important number of yearly publications superior to 73 papers in the last seven years. This number largely exceeds the number of yearly publications of most of the predatory biomedical research journals in Nigeria having an average of 29.1 scholarly publications per year [3]. This demonstrates that predatory behaviors cannot substitute the effect of good journal governance on enhancing the number of research outputs of local journals. As well, the number of yearly publications of the Moroccan Journal of Chemistry significantly exceeds the one of multiple national research journals in South Africa where traditions of maintaining research venues are more important [2]. This confirms the usefulness of having an open-access publication model where scientists can share their research findings free of charge.

**Fig. 2:**
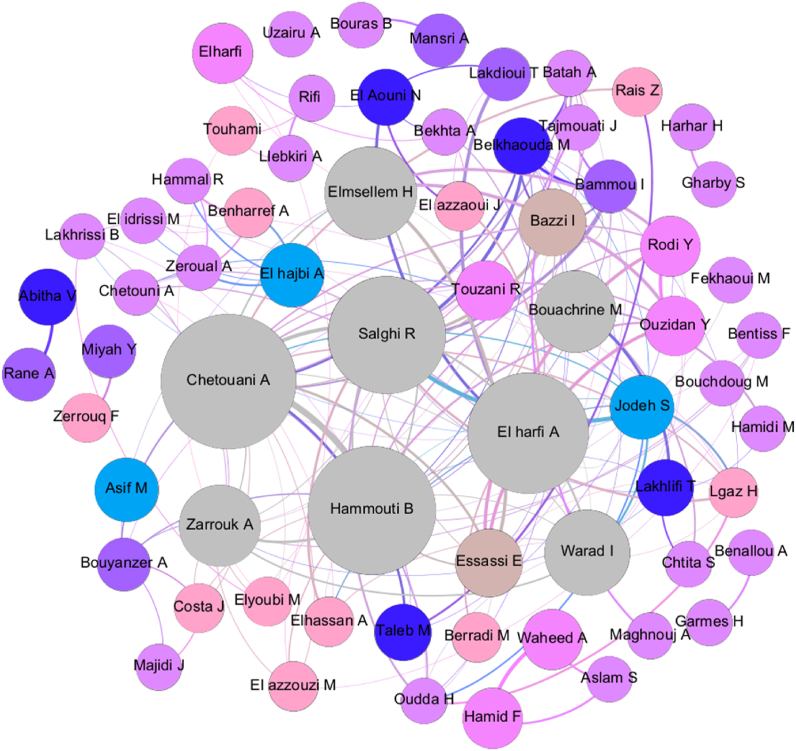
Collaboration network of the authors of the scholarly publications in the Moroccan Journal of Chemistry between 2013 and 2021. Grey nodes are the ones having the most links with other nodes. The blue-purple nodes are the ones having rare links with other nodes. Voluminous nodes are the ones having the best total link strength and small nodes are the ones having the smallest total link strength. Thick edges correspond to the most common links between nodes. (For interpretation of the references to colour in this figure legend, the reader is referred to the Web version of this article.)

When assessing the author collaboration network of the journal (Fig. 2), we found that there is an important tendency for collaboration between Moroccan scientists in the journal resulting in the creation of a cluster of over 40 chemical researchers mostly working in local scientific institutions. This cluster is led by eight productive research scientists including *Ahmed Chetouani* (Editor, 30 papers), *Belkheir Hammouti* (Founder and Editor-in-Chief, 27 papers), *Rachid Salghi* (25 papers), *Ahmed El Harfi* (25 papers)*, Hicham Elmsellem* (18 papers), *Mohammed Bouachrine* (16 papers), *Ismail Warad* (From Palestine, 16 papers), and *Abdelkader Zarrouk* (15 papers). This reveals that the Moroccan Journal of Chemistry is mainly used as a forum for local scientists to develop and discuss their large nationwide scholarly project proposals and initiatives by providing preliminary findings and upcoming research directions. This effect has been confirmed in several developing countries like Brazil where national journals contributed to the growth of local research [29]. The comparison of the main authors of the publications of the journal to the list of the most productive chemical scientists in Morocco for the period 2014–2017 identified that four of the eight main authors of the Moroccan Journal of Chemistry were among the most productive chemical scientists in Morocco between 2014 and 2017 as shown in Table 1. These four authors are part of the Editorial Board of the journal and their reputation can be consequently behind the attraction of the local chemical community to publish in this venue. This confirms in part that journals publish a large set of high-quality papers only when the editorial board is homogeneous and includes multiple respected editors [30]. The analysis of the list of the most productive chemical researchers in Morocco from 2018 to 2021 found the appearance of new highly productive scientists (Table 1) that successfully published research papers in the Moroccan Journal of Chemistry. This shows that the journal has contributed to stimulating the involvement of several local scientists (Table 1, in Italic) and foreign collaborators (*I. Warad*) in chemical research in Morocco. The journal can consequently serve as a tool to mature research careers through interaction with local scientists. This fact can explain the huge gap in productivity and citation between local scientists and researchers working abroad [31] by the lack of confirmed local journals in the Maghreb and the existence of long-term customs of national journal publishing in Europe and North America.

**Table 1:** Ten most productive scientists of Moroccan chemical research for the periods 2014–2017 and 2018–2021 according to the Web of Science Core Collection. Foreign collaborators are included. The main authors of the Moroccan Journal of Chemistry are in Bold. Local authors of the Moroccan Journal of Chemistry making their entry to the top ten most productive scientists after 2017 are in Italic.

2014–2017			2018–2021		
Rank	Scientist	PUB	Rank	Scientists	PUB
1	**B. Hammouti**	45	1	**A. Zarrouk**	115
2	T. Ben Hadda	35	2	**I. Warad**	84
3	**R. Salghi**	32	3	F. Benhiba	55
4	**M. Bouachrine**	27	4	**M. Bouachrine**	54
5	S. Radi	27	5	A. Amine	50
6	A. Amine	26	6	*H. Lgaz*	49
7	Y. N. Mabkhout	26	7	*E. Essassi*	46
8	A. Benyoussef	25	8	*B. Lakhrissi*	45
9	**A. Zarrouk**	23	9	J. T. Magne	42
10	Three scientists	20	10	**R. Salghi**	39

**Fig. 3:**
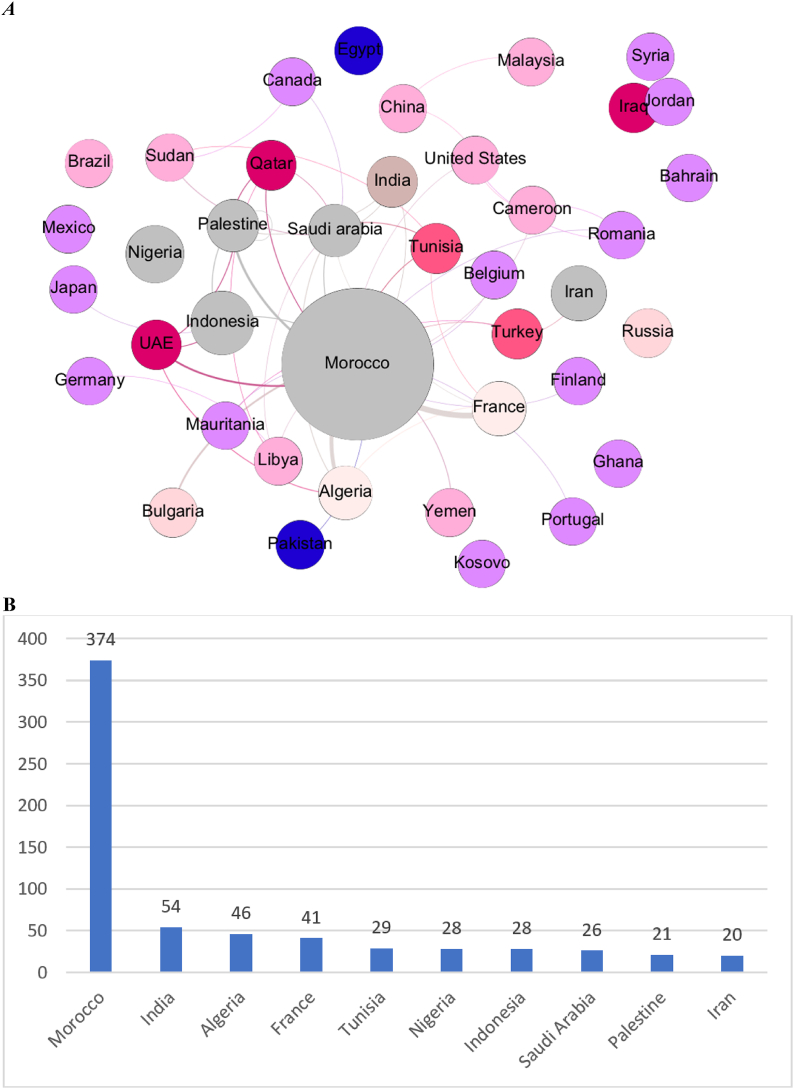
The contributions of countries to the Moroccan Journal of Chemistry: A) Collaboration network of the countries publishing research papers in the Moroccan Journal of Chemistry between 2013 and 2021. Grey nodes are the ones having the most links with other nodes. The blue-purple nodes are the ones having rare links with other nodes. Voluminous nodes are the ones having the best total link strength and small nodes are the ones having the smallest total link strength. Thick edges correspond to the most common links between nodes. B) The ten most productive countries in the Moroccan Journal of Chemistry. (For interpretation of the references to colour in this figure legend, the reader is referred to the Web version of this article.)

**Fig. 4:**
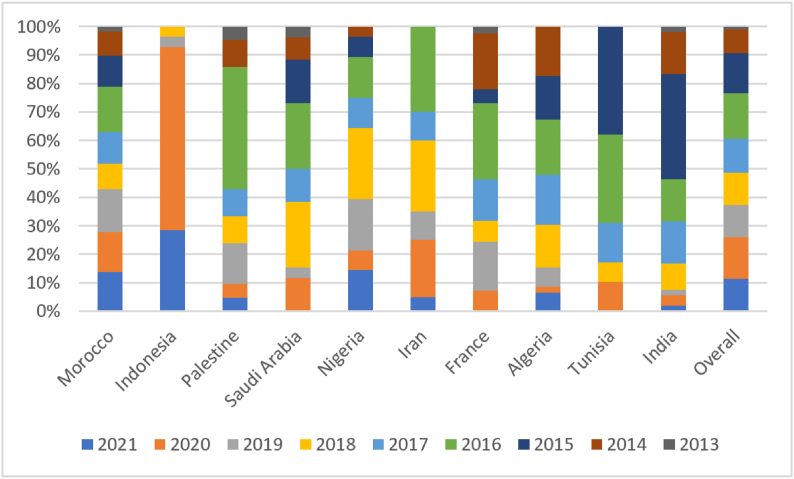
Yearly distribution of the scholarly contributions of the most published countries in the Moroccan Journal of Chemistry.

The significant existence of a foreign scientist in Moroccan chemical research following its scholarly contribution to the Moroccan Journal of Chemistry (*I. Warad*) led us to verify whether research collaborations at the scale of the journal can cause significant changes in the country collaboration patterns of Moroccan chemical research. When tracing the collaboration network between the published countries in the Moroccan Journal of Chemistry, we unsurprisingly found that Morocco was the most published country in the journal as shown in Fig. 3A and B with a rate of 58.3% (374 out of 641 publications). This confirms previous findings regarding the existence of publication bias for home countries in national scholarly journals specialized in exact sciences [32] and social sciences [33]. Yet, the rate of 41.7% of the publications not issued by local Moroccan scientists proves that the journal is not restricted to the local community and that it is open to host research from all over the world, proving the success of the journal’s internationalization. However, what is significant is the moderate weight of France in the collaboration network of the journal (Fig. 3A) despite being the main country that dominates chemical research collaborations with Morocco as shown in Table 2. When seeing the rate of contributions of France to Moroccan chemical research (Table 3), it is clear that it largely decreased from 32.7% (348 out of 1063 publications) for 2014–2017 to 21.2% (564 out of 2657 publications) for 2018–2021. When comparing this result with the number of scholarly publications of France in the Moroccan Journal of Chemistry (Fig. 3B), we find that France only contributed 6.3% (41 out of 641 publications) of the output of the journal, meaning that the nationwide research journal probably catalyzed the change of the collaboration policy of local chemical scientists. This does not go in line with older evidence proving that the nations having historical and scientific bonds with the country of origin of a national journal tend to be more represented in that venue [32]. Another considerable finding from the collaboration network of the published countries in the Moroccan Journal of Chemistry is the existence of countries that do not have collaboration traditions with Moroccan institutions and that are not featured in the list of the main countries collaborating with Morocco from 2014 to 2017 in chemical research as shown in Table 2. These countries particularly include Asian countries (e.g., *Palestine* and *Indonesia*) as shown in Fig. 3A. Some of these countries have surprisingly become among the main collaborators of Morocco in chemical research since 2018 (*China*, *Qatar*, *South Korea*, and *Palestine*). The increasing occurrence of *Saudi Arabia*, *Turkey*, the *United States of America*, and *India* in Moroccan chemical research (Bold in Table 2) can also be a direct result of the contribution to the Moroccan Journal of Chemistry as these countries are among the most weighted ones in the collaboration network of the journal (Fig. 3A), meaning that these countries have probably incubated their collaboration efforts with Morocco in the *Moroccan Journal of Chemistry*. This is mainly confirmed for Palestine and India which have a significant peak of publication in the Moroccan Journal of Chemistry before 2017 as shown in Fig. 4. The fact that Indonesia is not featured in the list of the main research collaborators of Moroccan in chemical scholarly research (Table 2) is mainly due to the occurrence of its peak of publication in the *Moroccan Journal of Chemistry* in 2020 (Fig. 4). This confirms that national journals can serve as incubators for establishing new international collaborations leading to the internationalization of national research communities. Preliminary outputs are published in local journals to scale up the results of the collaboration to meet national priorities and research standards [34]. For the context of Morocco, these research priorities involve the enhancement of life quality, socio-cultural development, knowledge, preservation and improvement of natural resources, information science and technology, agriculture in challenging conditions, corporate competitiveness and innovation, risk management, biotechnology, and basic research [35 p. 154]. Most of these topics can be developed using chemistry research projects.

**Table 2:** Ten most collaborative countries of Moroccan chemical research for the periods 2014–2017 and 2018–2021 according to the Web of Science Core Collection. Countries that had better collaboration rankings between 2018 and 2021 are in bold.

2014–2017			2018–2021		
Rank	Scientist	PUB	Rank	Scientists	PUB
1	France	348	1	France	564
2	Spain	131	2	**Saudi Arabia**	245
3	Saudi Arabia	67	3	Spain	189
4	Algeria	47	4	**Turkey**	119
5	Italy	41	5	**United States**	117
6	Germany	41	6	**India**	103
7	United States	36	7	**China**	91
8	Tunisia	33	8	Italy	88
9	Belgium	31	9	Belgium	73
10	Egypt	28	10	Algeria	72
11	Portugal	26	11	**Qatar**	72
12	Pakistan	26	12	Egypt	70
13	India	23	13	Tunisia	68
14	Canada	16	14	Germany	66
15	Turkey	15	15	**South Korea**	62
16	Two countries	14	16	**Palestine**	62

**Table 3:** Nineteen best-represented Web of Sciences Categories of Moroccan chemical research for the periods 2014–2017 and 2018–2021 according to Web of Science Core Collection.

2014–2017			2018–2021		
Rank	Research Area	PUB	Rank	Research Area	PUB
1	Chemistry Multidisciplinary	403	1	Chemistry Multidisciplinary	1087
2	Chemistry Physical	383	2	Chemistry Physical	972
3	Materials Science Multidisciplinary	144	3	Materials Science Multidisciplinary	287
4	Chemistry Analytical	112	4	Chemistry Analytical	267
5	Chemistry Applied	92	5	Biochemistry Molecular Biology	225
6	Chemistry Organic	90	6	Physics Atomic Molecular Chemical	217
7	Physics Atomic Molecular Chemical	74	7	Physics Applied	214
8	Electrochemistry	67	8	Chemistry Applied	197

**Fig. 5:**
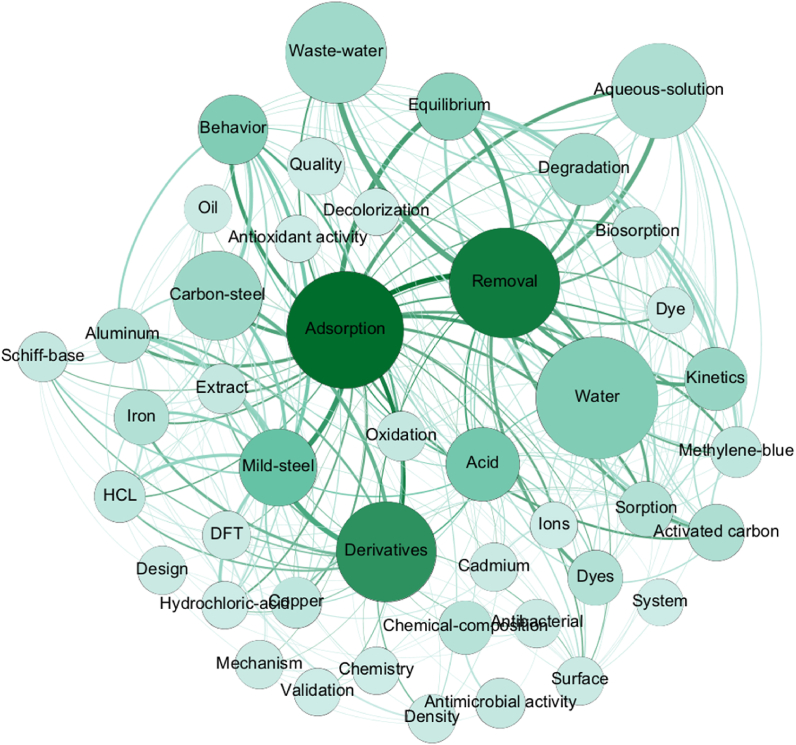
Co-occurrence Network of the Keywords Plus of the research papers published in the Moroccan Journal of Chemistry between 2013 and 2021. Dark green nodes are the ones having the most links with other nodes. The light green nodes are the ones having rare links with other nodes. Voluminous nodes are the ones having the best total link strength and small nodes are the ones having the smallest total link strength. Thick edges correspond to the most common links between nodes. (For interpretation of the references to colour in this figure legend, the reader is referred to the Web version of this article.)

When analyzing the Keywords Plus of the scholarly publications of the Moroccan Journal of Chemistry, we found that most of them are related to Applied Chemistry, Physical Chemistry, Multidisciplinary Materials Science, and Analytical Chemistry as shown in Fig. 5. Most of the papers deal with water processing (*waste-water* and *water*), food processing (*oil* and *antioxidant activity*), and alloys and compounds (*iron*, *aluminum*, *mild-steel*, *ions*, *copper*, and *cadmium*) using a variety of techniques such as chemical reactions on aqueous solutions (*aqueous-solution*, *Schiff-base*, *methylene-blue*, and *hydrochloric acid*), computational methods (*DFT*), physical methods (*kinetics* and *sorption*), and materials (*activated carbon*). This confirms previous findings about the growth of research in Morocco about water resources and management [36]. Effectively, ensuring water quality for Moroccan citizens is currently a state priority. Consequently, research affecting the situation in this context is highly encouraged by the Moroccan government contributing to the development of Moroccan research on water processing [36]. This also attests to the assumption that national journals tend to address relevant topics at the local scale by contrast to international journals that are mainly interested in global matters [34]. The availability of Moroccan chemical research related to materials is mainly explained by the existence of various productive local scientists working on the matter [37]. Some of these scientists are identified in Fig. 1 and seem to have a considerable research history and output stability over the years [37]. This can be verified through the analysis of the Web of Science Categories of Moroccan chemical research as shown in Table 3 where *Multidisciplinary Chemistry*, *Physical Chemistry*, and *Multidisciplinary Materials Science* are the main subfields of Chemistry in Moroccan research.

## 5 Conclusion

In this research paper, we analyzed the outputs of the *Moroccan Journal of Chemistry* between 2013 and 2021 through the assessment of their bibliographic data as revealed by the *Web of Science Core Collection* using a variety of tools including descriptive statistics and network analysis. Then, we compared the results of our study to the landscape of Moroccan chemical research for providing the impact of the development and internationalization of the Moroccan Journal of Chemistry on the local chemical research community. We found that the Moroccan Journal of Chemistry has successfully evolved thanks to its free-of-charge open-access research policy. On the one hand, the journal has successfully evocated multiple topics ranging from water management to materials processing within the framework of the Moroccan national research priorities. On the other hand, the journal has encouraged local and international research networking by letting the journal a forum for discussing and enhancing preliminary Moroccan research initiatives in Chemistry. The journal serves as an incubator to develop research on novel topics that have been evocated before by local chemical research. As well, this journal is very useful to launch new research collaborations with untraditional target countries such as Asian (e.g., *Palestine, China, India*, and *Indonesia*) and African (e.g., *Nigeria*) countries freeing the host nation (i.e., *Morocco*) from its long-term dependency on French scholarly institutions, allowing the internationalization of the *Moroccan Journal of Chemistry*.

This internationalization induces the development of an international collaboration network for local chemical research, leading to an increase in the topical coverage and diversity of the local chemical research outputs that cover a variety of topics corresponding to local and international contexts. The high level of collaboration between Moroccan and international researchers guarantees greater visibility and impact for researchers. As well, we identified that the journal serves as a model to develop South-South cooperation and collaborative partnerships between countries in the global south for mutual benefit and development. To develop South-South cooperation beyond French-speaking countries, it is important to identify common challenges and opportunities, establish clear communication and dialogue channels, and pursue mutually beneficial initiatives. This can involve promoting scientific exchange programs and facilitating technology transfers. It is also important to build relationships with non-French-speaking countries to foster international collaboration.

## 6 Limitations and future directions

Despite the significance of our findings, they do not cover multiple aspects of journal internationalization, particularly citation, readership, and editorial board internationalization. Furthermore, they are restricted to the analysis of a single OJS-based national journal even though Morocco issues 102 free-of-charge open-access journals. As a future direction of this research work, we look forward to verifying the effect of national journals on the research outputs of their host countries, particularly in the Global South nations. We also envision studying the effect of the free-of-charge open-access policy of the nationwide journals on the internationalization of their editorial boards, their readership, and their citations.

## Author contribution statement

All authors listed have significantly contributed to the development and the writing of this article.

## Data availability statement

Data associated with this study has been deposited at www.edream.ma.

## Additional information

No additional information is available for this paper.

## Declaration of competing interest

The authors declare that they have no known competing financial interests or personal relationships that could have appeared to influence the work reported in this paper

## Footnotes

1 Further information can be found at https://revues.imist.ma/index.php/morjchem/about.

2 Statistical data about the geographical distribution of OJS-based journals is available at https://rstudio-pubs-static.s3.amazonaws.com/757818_3efc455fb6b54d949429d7a6fb9196ac.html#active-ojs-version-2-installation-urls.

3 Query: Morocco (Address) AND Chemistry (Research Area).
